# An ANNEXIN-Like Protein from the Cereal Cyst Nematode *Heterodera avenae* Suppresses Plant Defense

**DOI:** 10.1371/journal.pone.0122256

**Published:** 2015-04-07

**Authors:** Changlong Chen, Shusen Liu, Qian Liu, Junhai Niu, Pei Liu, Jianlong Zhao, Heng Jian

**Affiliations:** 1 Department of Plant Pathology, China Agricultural University, Beijing, China; 2 Institute of Plant Protection and Agro-products Safety, Anhui Academy of Agricultural Sciences, Hefei, Anhui, China; 3 Tropical Crops Genetic Resources Institute, Chinese Academy of Tropical Agricultural Sciences, Danzhou, Hainan, China; James Hutton Institute, UNITED KINGDOM

## Abstract

Parasitism genes encoding secreted effector proteins of plant-parasitic nematodes play important roles in facilitating parasitism. An annexin-like gene was isolated from the cereal cyst nematode *Heterodera avenae* (termed *Ha-annexin*) and had high similarity to *annexin 2*, which encodes a secreted protein of *Globodera pallida*. *Ha-annexin* encodes a predicted 326 amino acid protein containing four conserved annexin domains. Southern blotting revealed that there are at least two homologies in the *H*. *avenae* genome. *Ha-annexin* transcripts were expressed within the subventral gland cells of the pre-parasitic second-stage juveniles by *in situ* hybridization. Additionally, expression of these transcripts were relatively higher in the parasitic second-stage juveniles by quantitative real-time RT-PCR analysis, coinciding with the time when feeding cell formation is initiated. Knockdown of *Ha-annexin* by method of barley stripe mosaic virus-based host-induced gene silencing (BSMV-HIGS) caused impaired nematode infections at 7 dpi and reduced females at 40 dpi, indicating important roles of the gene in parasitism at least in early stage *in vivo*. Transiently expression of Ha-ANNEXIN in onion epidermal cells and *Nicotiana benthamiana* leaf cells showed the whole cell-localization. Using transient expression assays in *N*. *benthamiana*, we found that Ha-ANNEXIN could suppress programmed cell death triggered by the pro-apoptotic mouse protein BAX and the induction of marker genes of PAMP-triggered immunity (PTI) in *N*. *benthamiana*. In addition, Ha-ANNEXIN targeted a point in the mitogen-activated protein kinase (MAPK) signaling pathway downstream of two kinases MKK1 and NPK1 in *N*. *benthamiana*.

## Introduction

*Heterodera avenae* is one of the most important cereal cyst nematodes (CCNs) in the world. This pathogen is distributed worldwide on cereal crops and occurs in approximately 80% of the total cereal growing areas in China [1]. Additionally, *H*. *avenae* causes substantial economic yield losses; in some wheat fields, the losses caused by this nematode can range from 30 to 100% [2, 3].

*H*. *avenae* is an obligate sedentary plant parasitic nematode that invades the roots of wheat and related cereals in the subfamily Pooideae. The second-stage juveniles (J2s) penetrate the root tip and migrate intracellularly through the cortex to the vascular cylinder, where the nematode inserts its stylet into a selected parenchyma cell and induces its transformation into a feeding site. The stylet is used to deliver secretions referred to as “effectors” into root tissue, which facilitates plant parasitism.

The identification of genes encoding candidate effector proteins has gained increasing attention in molecular plant nematology research in the last two decades. However, only a few effectors have been reported in *H*. *avenae*. Three β-1,4-endoglucanase genes, whose transcripts accumulate specifically in the two subventral gland cells of *H*. *avenae*, have been identified and suggested to play a crucial role in plant cell wall-degradation during the penetration and migration of nematodes in the host roots [4, 5]. A new expansin gene (*Ha-expb1*) expressed in the subventral glands of *H*. *avenae* was cloned and has been suggested to play a role in the early parasitic-stage process, most likely aiding migration within the plant [6]. Cathepsin S-like cysteine proteinase of *H*. *avenae* was isolated, and its plausible mode of interaction was illustrated by docking analysis. Additionally, qRT-PCR analysis has suggested that this proteinase has an important role in both pre-parasitic and parasitic stages of the nematode life cycle [7]. Undoubtedly, these findings are far from unraveling the parasitism mechanism of *H*. *avenae*. Transcriptome data on *H*. *avenae* reported this year provide an opportunity to identify new effectors that are specifically involved in *H*. *avenae*-host interactions [8].

It has been reported that annexins play important roles in animals and plants, including exocytosis, actin binding, peroxidase activity, callose synthase regulation, ion transport and the ability to link Ca^2+^, redox and lipid signaling to coordinate development with responses to the biotic and abiotic environment [9, 10]. The diverse family of proteins in plant parasitic nematodes has also been examined, and there have been some important findings. In 2001, an annexin named gp-nex was identified from *Globodera pallida* and was found to be immunolocalized in the amphids, genital primordium and constraining muscles above and below the metacorpus pump chamber [11]. In 2003, *Hg4F01*, a gene similar to annexins in the nematode *Caenorhabditis elegans*, was isolated from the parasitome of *Heterodera glycines*, which is expressed exclusively in the dorsal esophageal gland cell of the parasitic stages [12]. In *Heterodera schachtii*, the molecular function of annexin involved in the host-nematode interaction was described. The expression of annexin in wild-type *Arabidopsis* promoted hyper-susceptibility to *H*. *schachtii* infection, and in an *AnnAt1*(*annexin-1* of *Arabidopsis*) mutant reverted mutant sensitivity to 75 mM NaCl (high salt condition), suggesting a similar function to *AnnAt1* in the stress response by plant cells. Additionally, yeast two-hybrid assays showed that annexin interacted with an oxidoreductase member of the 20 G Fe (II) oxygenase family that is linked to host defense and stress response. All of this evidence suggests that annexin from *H*. *schachtii* may mimic plant annexin function to modulate host defense responses to promote parasitism [13].

Recently, additional reports describing the suppression of plant defense by nematode effectors have emerged [14–18]. The plant defense system that responds to infection by pathogens consists of two overlapping branches: PAMP-triggered immunity (PTI) and effector-triggered immunity (ETI). PTI responds to conserved microbial- or pathogen-associated molecular patterns (MAMPs or PAMPs, respectively). ETI is triggered by the recognition of pathogen effector molecules by the plant defense system. ETI usually has a hypersensitive cell death response (HR) at the infection site, as does PTI in certain cases. The programmed cell death (PCD) triggered in plants by the pro-apoptotic mouse protein BAX physiologically resembles that associated with the defense-related HR. As a result, the ability to suppress BAX-triggered PCD (BT-PCD) has been proven a valuable initial screening tool for pathogen effectors capable of suppressing defense-associated PCD [19–22]. With regard to PTI, the increased expression of defense-related genes is one of its phenotypes. Plant mitogen-activated protein kinase (MAPK) cascades also play a pivotal role in the PTI signaling pathway by transducing signals from pattern recognition receptors (PRRs) to downstream components [23–27]. MAPK cascades consist of at least three protein kinases: a MAPK kinase kinase phosphorylates and activates a MAPK kinase, which in turn activates a MAPK by phosphorylation [22]. MKK1 encodes a MAPK kinase, and NPK1 encodes a MAPK kinase kinase that functions to transduce PAMP-triggered signals [22, 28–31]. Genes encoding full-length MKK1 and the N terminus of NPK1 (residues 1 to 373; NPK1^Nt^) could trigger PCD when introduced by agroinfiltration into *Nicotiana benthamiana*, and Wang found that Avh238^P7076^, an effector of *Phytophthora sojae*, could suppress the PCD triggered by both MAPKs, suggesting that this effector acted at a point in the signaling pathway downstream of the two kinases [22].

In this study, we report the identification and certain functional characteristics of an annexin-like gene from the cereal cyst nematode *H*. *avenae* that most likely suppresses plant immunity to facilitate parasitism.

## Materials and Methods

### Nematodes

*Heterodera avenae* was propagated on wheat (*Triticum aestivum* cv. Aikang 58) in an artificial environment. Embryo eggs were pipetted from crushed newly formed cysts. Infective second-stage juveniles were collected by hatching cysts at 15°C after at least 4 weeks incubation at 4°C. To obtain parasitic life stages, infected wheat roots were obtained at different days after inoculation, washed with tap water, cut into sections and digested at 28°C with 160 rpm in a 6% cellulose (Sinopharm Chemical Reagent Beijing Co., Ltd., China) water solution for 12 h. After digestion, the roots were placed on a sieve set and crushed by rapid-flow water to release nematodes onto the sieve. Adult females were directly washed from the root surface.

### Cloning of the *Ha-annexin* cDNA and gDNA sequence

mRNA was extracted from cysts of *H*. *avenae* using a Dynabeads mRNA DIRECT Kit (Invitrogen, USA) and cDNA was synthesized using reverse transcriptase SuperScript III (Invitrogen, USA) according to the manufacturer’s instructions. Part of the *Ha-annexin* gene was PCR-amplified from the cDNA using 29D09-F/29D09-R primer pairs (S1 Table), which were designed according to the clone *29D09* (GenBank Accession AF500016.1), a *Heterodera glycines* homologue originally reported to be a pioneer from the parasitome [12]. The PCR product was cloned into the pMD18-T vector (Takara, Japan), transformed into DH5α-competent cells, and sequenced. To obtain the full-length cDNA of the *Ha-annexin* gene, 3’ RACE and 5’ RACE procedures were conducted using a SMART RACE cDNA Amplification Kit (Clontech, USA) and a 5´ RACE System for Rapid Amplification of cDNA Ends, Version 2.0 (Invitrogen, USA), respectively. All of the procedures were conducted according to the user manuals with additional self-designed primers. For the 3’ RACE, the primers annexin-S1 (S1 Table) and UPM (kit available) were used to amplify the fragment of the 3’ end. For the 5’ RACE, the primer annexin-R (S1 Table) was used to synthesize the first-strand cDNA, and the primers annexin-A1 (S1 Table) and AAP (kit available) were used to amplify the fragment of the 5’ end for the first PCR followed by nested amplification using primers annexin-A2 (S1 Table) and UAP (kit available) to obtain the fragment of the 5’ end. Both 3’ and 5’ ends were sequenced and assembled with the sequence of the initial cloned fragment to obtain the full-length cDNA sequence of *Ha-annexin*.

For cloning the gDNA sequence of *Ha-annexin*, the primers annexinQCF4/annexinQCR4 (S1 Table) were used, and the product was also sequenced.

### Bioinformation analysis

The predicted protein sequence of Ha-ANNEXIN was blasted for closed protein using BLASTP searching of the protein databases of the National Center for Biotechnology Information (NCBI) (http://blast.ncbi.nlm.nih.gov/Blast.cgi). Prediction of a signal peptide for secretion was performed using Signal P 4.0 (http://www.cbs.dtu.dk/services/SignalP/). Multiple amino acid sequence alignment of annexins from *H*. *avenae*, *G*. *pallida*, *Bursaphelenchus xylophilus*, *H*. *schachtii* and *H*. *glycines* was conducted using DNAMAN V6, and conserved domains were searched using the Conserved Domain Search Service (CD Search) of the NCBI (http://www.ncbi.nlm.nih.gov/Structure/cdd/wrpsb.cgi).

### Southern blot hybridization

*H*. *avenae* genomic DNA was extracted using a QIAamp DNA Micro Kit (Qiagen, Germany). Five micrograms of genomic DNA was digested overnight at 37°C with *Bam*HI or *Eco*RI (New England Biolabs, USA), separated by 0.8% agarose gel electrophoresis and transferred onto a Hybond-N membrane (GE Healthcare, USA) using standard protocols [32]. The membrane was hybridized with the *Ha-annexin*-CDS DNA probe, which was random primed labeled with Digoxigenin-11-dUTP using DIG-High Prime (Roche). Hybridization (at 42°C) and detection were performed following the instruction manual of the DIG High Prime DNA Labeling and Detection Starter Kit I (Roche, USA).

### mRNA *in situ* hybridization

*In situ* hybridization was performed as previously described [33] but with the hybridization temperature adjusted to 50°C. The specific primers anne-qRT-S and anne-qRT-A (S1 Table) were used to synthesize digoxigenin (DIG)-labeled sense (control) and antisense cDNA probes (Roche, USA) by asymmetric PCR [34].

### Developmental expression analysis

Total RNA of different life stages of *H*. *avenae* was extracted using the RNeasy Plus Micro Kit (Qiagen, Germany), which includes gDNA eliminator columns to remove DNA. cDNA was prepared according to the instructions for the QuantiTect Whole Transcriptome Kit (Qiagen, Germany), which can provide sufficient cDNA for gene expression analysis by quantitative real-time PCR (qPCR). A SYBR Green assay was used to quantify the expression of *Ha-annexin* throughout the nematode life cycle. qPCR was performed using a SYBR Premix Ex Taq (TaKaRa, Japan) in an ABI Prism 7000 instrument (Applied Biosystems, USA), with primers anne-qRT-S/anne-qRT-A and GAPDH-qS1/ GAPDH-qAS1 designed (S1 Table), respectively, from the *Ha-annexin* coding sequence and an endogenous control gene *GAPDH-1* (sequence from internal data). Triplicate PCR reactions for each cDNA sample were completed, and the assay consisted of three technical replicates. Data were analyzed using the 2^-ΔΔCt^ method [35].

### Subcellular localization *in planta*

#### Subcellular localization in onion

*Ha-annexin* ORF sequences (without a stop codon) were amplified using primers ann-*Xba* I-S and ann-*Xho* I-AS (S1 Table) for cloning into the pUC35SGFP vector to generate the 35S:ANNEXIN:GFP construct by the method of digestion and connection. Each construct plasmid (4–5 μg/shot) was delivered into onion epidermal cells through biolistic bombardment by vacuumizing to 27–28 in. Hg using a PDS1000/He system (Biolistic Particle Delivery System, Bio-Rad, USA). After bombardment, epidermal peels were incubated at 25°C for 24 h in the dark. The subcellular localization of the fused proteins was visualized using laser confocal fluorescence microscopy (Nikon Eclipse TE300, Nikon, Japan) at an excitation wavelength of 488 nm.

#### Subcellular localization in *N*. *benthamiana*

*Ha-annexin* was constructed into pCamv35SGFP vector with GFP fused at the C-terminal, according to the user manual of the In-Fusion HD Cloning Kit (Clontech, USA), with the primers ann-if-pCam35SGFP-S1/AS1 (S1 Table). The construct was confirmed by sequencing and transformed into *Agrobacterium tumefaciens* strain EHA105. *N*. *benthamiana* plants were grown in a growth room for 4 weeks at approximately 25°C with a 14 h light/10 h dark cycle. For infiltration into leaves, recombinant strains of *A*. *tumefaciens* of pCamv35SGFP-annexin and pCamv35SGFP-p19 (retained by the lab; p19, a RNA silencing suppressor) were cultured, collected, washed three times and resuspended with infiltration buffer [10mM MgCl_2_ in 10 mM 2-(N-morpholino) ethanesulfonic acid (MES), pH 5.2, and 0.1 mM acetosyringone] to ~0.5 OD_600_, respectively. Then, 3:1 amounts of pCamv35SGFP-annexin and pCamv35SGFP-p19 were mixed, collected and resuspended to 1/2 of the total volume, before infiltrated to *N*. *benthamiana* leaves. After infiltration, *N*. *benthamiana* plants were incubated at 22°C for 48 h with the same light/dark cycle. pCamv35SGFP (retained by the lab) was used as a control. The subcellular localization was visualized as described in “Subcellular localization in onion”. For verification of intact of annexin-GFP fusion, western blotting was performed. A primary mouse anti-GFP monoclonal antibody in 1: 5000 dilution (Medical & Biological Laboratories, Japan), a goat anti-IgG, (mouse) pAb-HRP secondary antibody in 1: 5000 dilution (Medical & Biological Laboratories, Japan) and a DAB Kit (Beijing ComWin Biotech Co., Ltd., China) for color visualization were used for detecting the expression of annexin-GFP fusion and GFP control.

### Knockdown of *Ha-annexin* by BSMV-HIGS and infection assay

We conducted barley stripe mosaic virus (BSMV)-mediated gene silencing as previously described [36]. The three BSMV component vectors (pCaBS-α, pCaBS-β and pCaBS-γ) were used. Selected specific gene fragments of *Ha-annexin* and *eGFP* control, whose specificity was confirmed by being blasted to NCBI data and our un-published *H*. *avenae* transcriptome data, were amplified by PCR using the primer pairs annG3-LIC-pBS-S1/AS1 and eGFPG1-LIC-pBS-S1/AS1 (S1 Table), respectively, and constructed to pCaBS-γ. Inoculation of 300J2s of *H*. *avenae* to each plant was performed approximately 9 days after the tobacco sap inoculation of leaves of wheat (*T*. *aestivum* cv. Aikang 58). After nematode inoculation, wheat seedlings were placed in an illumination incubator at 21–22°C with a 16 h light/8 h dark cycle. The roots infected with nematode were stained using the sodium-hypochlorite-acid fuchsin method [37] at 7 dpi (days post nematode inoculation) and then were observed and counted under a microscope (n = 5), and females on plant at 40 dpi were also counted (n = 8). Juveniles inside root were separated by digestion with cellulose (as detailed in Section of “Nematodes”), and the expression level changes of *Ha-annexin* in nematodes recovered from wheat inoculated by BSMV:*annexin* compared to the blank negative control BSMV:00 and the negative control BSMV:*eGFP* were determined by qPCR. The experiment was repeated twice.

### **Cell**-**death suppression assay in *N*. *benthamiana***

*Ha-annexin* was constructed into PVX vector pGR107 with a flag-tag fused at the N-terminal, according to the user manual of the In-Fusion HD Cloning Kit (Clontech, USA), with the primers ann-if107f-S1 and ann-if107f-AS1 (S1 Table). *eGFP* was constructed into the vector with a flag-tag fused at the N-terminal by the method of digestion and connection using the primers *Sma*I-GFP-ORF and GFP-ORF-*Sal*I (S1 Table). The full-length *MKK1* and the N terminus of *NPK1* (residues 1 to 373; NPK1^Nt^) were constructed into pGR107 with an HA-tag, according to the user manual of the In-Fusion HD Cloning Kit (Clontech, USA), with the primer pairs MKK1-if-p107HA-S1/AS1 and NPK1(Nt)-if-p107HA-S1/AS1 (S1 Table), respectively. The constructs were confirmed by sequencing and transformed into *A*. *tumefaciens* strain GV3101. *N*. *benthamiana* plants were grown in a growth room for 4 to 6 weeks at approximately 25°C with a 14 h light/10 h dark cycle. Assays of the suppression of BT-PCD and M/NT-PCD (MKK1/NPK1 ^Nt-^triggered PCD) were performed as previously described [22], except that *A*. *tumefaciens* cells carrying the *Bax* and *MKK1/NP*K1 ^*Nt*^ gene were infiltrated only at 24 h and 16 h after the initial inoculation, respectively. The assays were repeated twice, with 3–7 plant replicates inoculated on three leaves each.

For verification of gene expression, reverse transcription-PCR (RT-PCR) or western blotting were performed. Total RNA and proteins were isolated from infiltrated parts of leaves of *N*. *benthamiana* using TRIzol Reagent (Invitrogen, USA). For RT-PCR, cDNA was synthesized as described in Section of “Cloning of the *Ha-annexin* cDNA and gDNA sequence” and PCR was performed using the gene-specific primers ann-if107f-S1/ ann-if107f-AS1, *Cla*I-Bax-ORF/ Bax-ORF-*Sal*I, and eGFP-F1/ eGFP-F2 (S1 Table) for *Ha-annexin*, *Bax* and *eGFP*, respectively. For western blotting, a primary rabbit anti-FLAG polyclonal antibody in 1: 6000 dilution (Cell Signaling Technology, USA), a goat anti-rabbit IgG, HRP-conjugated secondary antibody in 1: 3000 dilution (Beijing ComWin Biotech Co., Ltd., China) and a DAB Kit (Beijing ComWin Biotech Co., Ltd., China) for color visualization were used for detecting the expression of ANNEXIN and eGFP control, both fused with FLAG. A primary anti-Bax monoclonal antibody (Abcam, UK) in 1 mg/mL, a goat anti-IgG, (mouse) pAb-HRP secondary antibody in 1: 5000 dilution (Medical & Biological Laboratories, Japan) and a DAB Kit (Beijing ComWin Biotech Co., Ltd., China) for color visualization were used for detecting the expression of Bax.

Photos of phenotypes of infiltrated leaves of *N*. *benthamiana* were taken approximately 7 days after the last infiltration. The degree of PCD of Ha-ANNEXIN and control eGFP followed by BAX, MKK1 or NPK1^Nt^, referred to as the “Necrosis Index”, was scored on a ten-point scale according to the size of the necrotic area (grade 1 for 10% necrosis of the whole circle area, grade 2 for 20%, and so on). Necrosis Indices of ANNEXIN and eGFP were compared.

### PTI marker gene expression in *N*. *benthamiana*

The experiment was conducted as previously reported [17]. The expression of three marker genes—*NbPti5*, *NbAcre31* and *NbGras2*—of PTI [38], induced by flg22 (SciLight Biotechnology, China) or distilled water in *N*. *benthamiana* tissue infiltrated by *A*. *tumefaciens* cells carrying *annexin* or *eGFP* control, were evaluated by qPCR. Isolation of total RNA and cDNA synthesis was conducted in the same way as in the cell-death suppression assay. Gene expression was determined by qPCR as described in Section of “Developmental expression analysis”, and the reference gene *NbEF1α* [39] was used. The qPCR primers for *NbPti5*, *NbAcre31*, *NbGras2* and *NbEF1α* were NbPti5-F/R, NbAcre31-F/R, NbGras2-F/R and NbEF1α-F/R (S1 Table), respectively [38]. Two independent experiments were performed with triplicate PCR reactions each. The changes in marker gene expression in *N*. *benthamiana* tissues expressing ANNEXIN or eGFP were compared.

### Statistical analysis

Data difference of treatments was analyzed by a one-way ANOVA (Duncan test) or an independent-samples t-test performed in SPSS 13.0.

## Results

### Isolation and sequence analysis of the annexin-like gene from *H*. *avenae*

The full-length cDNA of *Ha-annexin* (GenBank Accession KJ562871) obtained by the 5’ and 3’ end amplification of cDNA is 1201 bp long, composed of a 981 bp open reading frame (ORF), a 63 bp 5’-untranslated region (5’-UTR) before the ATG initiation codon and a 157 bp 3’-UTR containing a polyA tail. The gDNA sequence of *Ha-annexin* (GenBank Accession KJ562872) lacks an intron.

**Fig 1:**
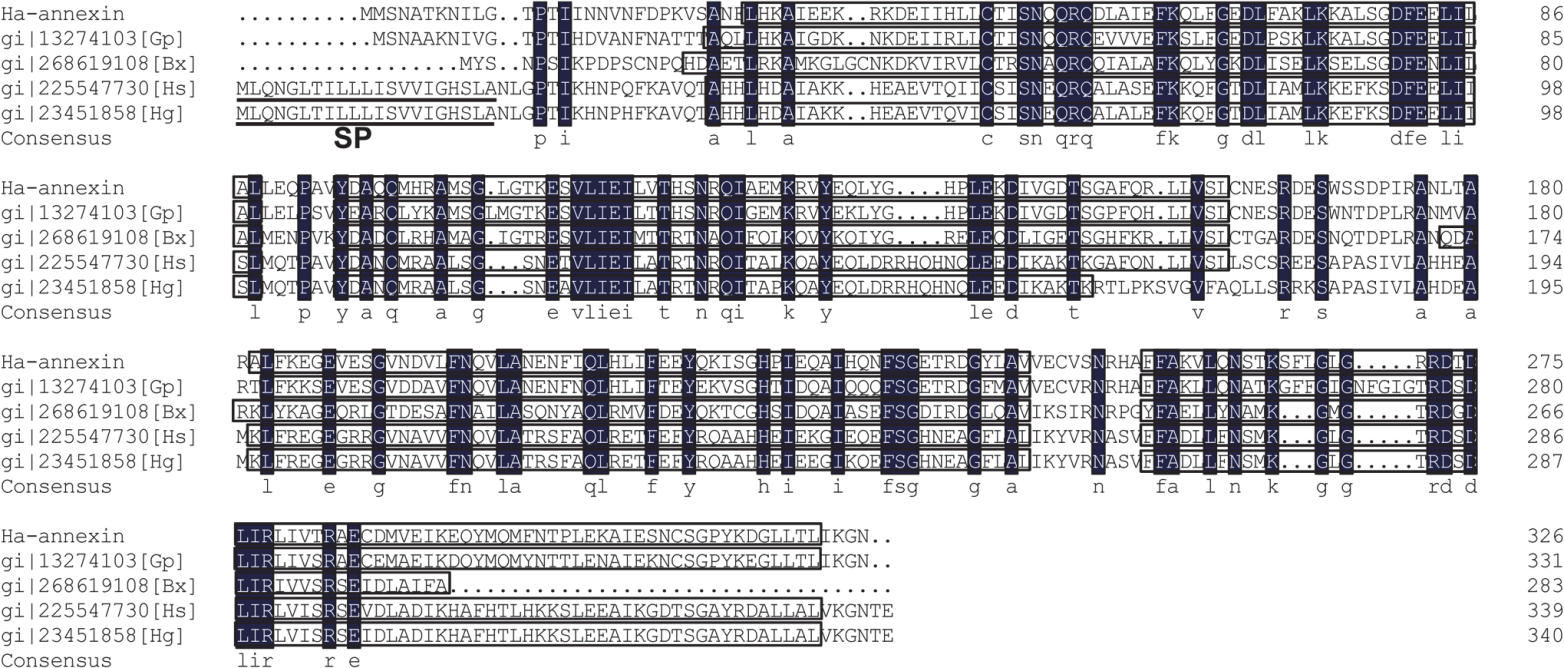
Multiple alignment of annexins from *Heterodera avenae* and some other plant-parasitic nematodes. In the consensus, residues in the black background are totally identical; boxed areas are four conserved annexin domains predicted by the NCBI Conserved Domain Search Service; signal peptides of *H*. *schachtii* and *H*. *glycines* analyzed by Signal P 4.0 are underlined. SP, signal peptide; Gp, *Globodera pallida*; Bx, *Bursaphelenchus xylophilus*; Hs, *H*. *schachtii*; Hg, *H*. *glycines*.

The protein encoded by *Ha-annexin* was predicted to have 326 amino acids and showed 76% identity to annexin 2, a secreted protein of *G*. *pallida* by using BLASTP. Similar to annexins from *G*. *pallida* and *B*. *xylophilus*, Ha-ANNEXIN had no secretion signal peptide at the N-terminal predicted by Signal P 4.0, but different from those of the plant-parasitic nematodes (PPNs) *H*. *schachtii* and *H*. *glycines*. These annexins from different PPNs all have four conserved annexin domains (Fig 1), which is typical of the annexin family of calcium and phospholipid binding proteins.

**Fig 2:**
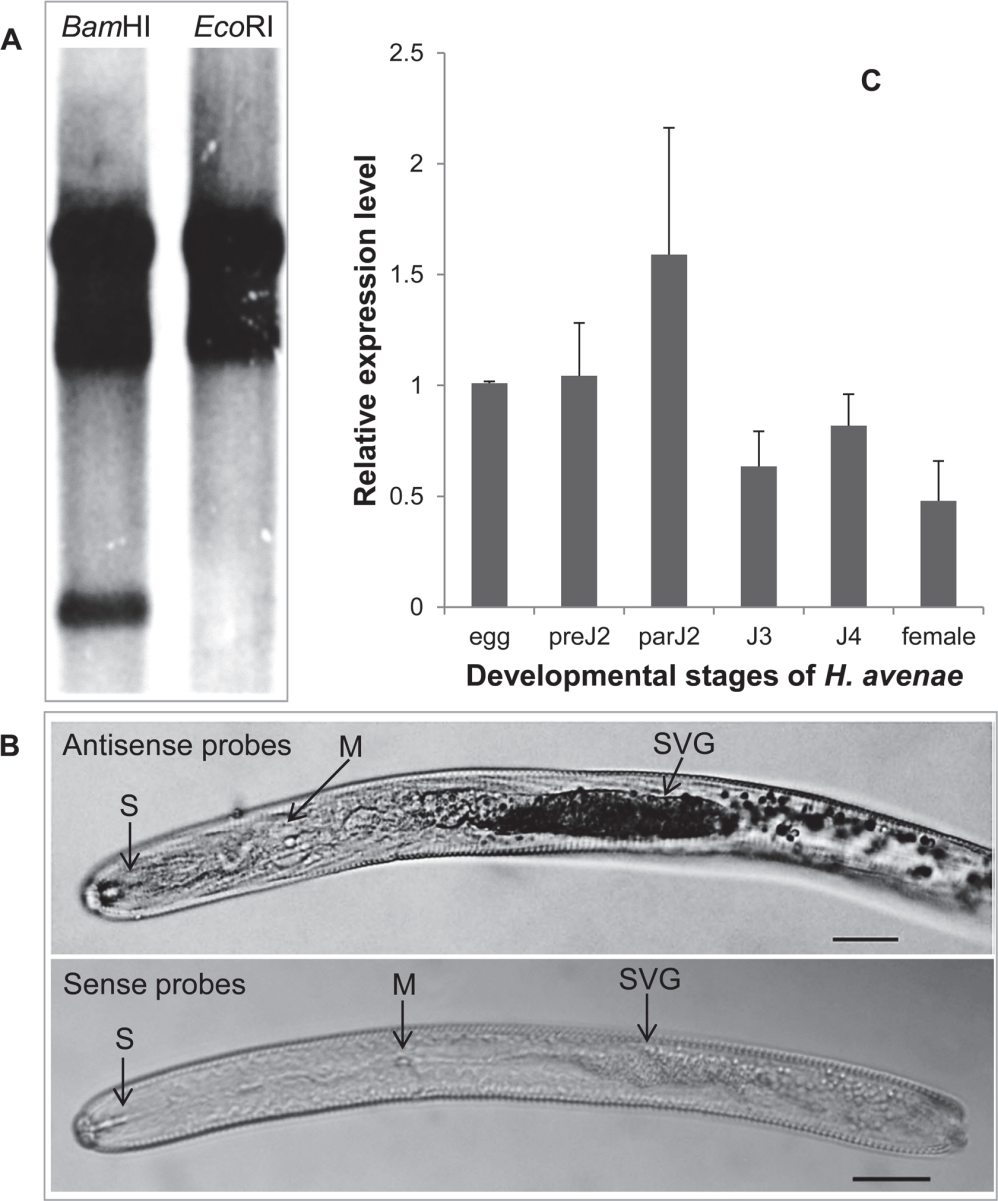
Southern blot, *in situ* hybridization and developmental expression pattern analysis of *Ha-annexin*. (A) Southern blot analysis of *Ha-annexin*. Genomic DNA from *H*. *avenae* was digested with *Bam*HI and *Eco*RI, respectively, and probed with the Dig-labeled CDS of *Ha-annexin*. They had 3 and 2 signal strips, respectively. (B) *In situ* hybridization of the *Ha-annexin* transcripts in pre-parasitic second-stage juveniles. Signal of antisense *Ha-annexin* DIG-Labeled cDNA probes localized within the subventral glands (SVGs), with sense probes as a negative control. The SVG, metacorpus (M), and stylet (S) are indicated with arrows. Scale bar = 20 μm. (C) Developmental expression pattern of *Ha-annexin*. The relative expression level of *Ha-annexin* was quantified using qPCR for six different *H*. *avenae* stages. The fold change values were calculated using the 2^-ΔΔCt^ method and presented as the change in mRNA level in various nematode developmental stages relative to that of egg. Each column represents the mean of 3 independent assays with standard deviation. preJ2: pre-parasitic second-stage juvenile; parJ2, J3 and J4: parasitic second-, third- and fourth-stage juvenile, respectively.

Southern blotting showed that DIG-labeled *Ha-annexin* probes hybridized to at least two DNA fragments on DNA gel blots (Fig 2A), which suggested that two or more members of an annexin gene family may exist in *H*. *avenae*.

### *Ha-annexin* expression is gland specific and relatively higher in parJ2

*In situ* hybridization was performed in pre-parasitic second-stage juveniles (preJ2s) of *H*. *avenae* to localize the expression of *Ha-annexin* transcripts. The hybridization signal of DIG-labeled antisense probes was observed in the subventral gland cells of *H*. *avenae*, and no signal was detected when using sense probes (Fig 2B).

The developmental expression pattern of *Ha-annexin* was determined by qPCR analysis for six developmental stages (egg; preJ2; parasitic second-, third- and fourth- stage juvenile (parJ2, J3, and J4, respectively); and adult female). The mean values of the three independent replicates were presented in Fig 2C, that is, the expression level of *Ha-annexin* transcripts in parJ2 was relatively higher than in other developmental stages.

### Ha-ANNEXIN is localized in the whole plant cell

**Fig 3:**
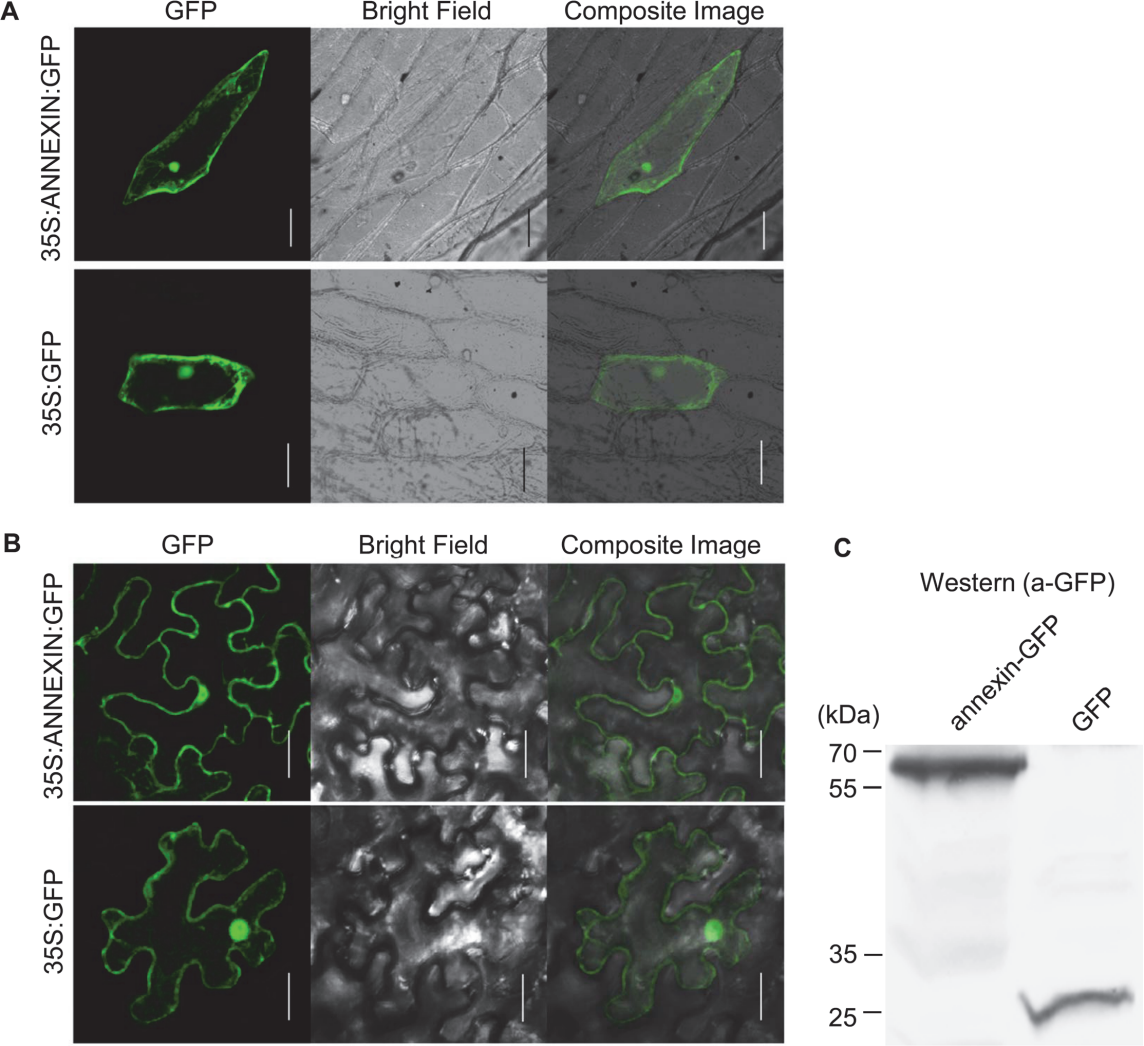
Subcellular localization of Ha-ANNEXIN in the plant cell. (A) pUC35S:ANNEXIN:GFP fusion construct and pUC35S:GFP control construct were transformed into onion epidermal cells by bombardment. Scale bar = 100 μm. (B) *Agrobacterium tumefaciens* cells carrying pCamv35S:ANNEXIN:GFP fusion and pCamv35S:GFP were transiently expressed in *Nicotiana benthamiana* cells. Scale bar = 20 μm. Western blotting of *N*. *benthamiana* leaves infiltrated with pCamv35SGFP-annexin showed expected size of annexin-GFP fusion, which is larger than GFP control. In both (A) and (B), GFP signals were observed in the whole transformed cells for annexin-GFP fusion, which is the same as GFP control.

Ha-ANNEXIN is expected to be delivered to the host cell through nematode stylets. To test where Ha-ANNEXIN localizes in plant cells, a protein transient expression assay was performed in both onion and *N*. *benthamiana*. The GFP signal was observed in the whole cell as the vector control (Fig 3), which indicated the whole cell-localization of Ha-ANNEXIN. Western blotting of *N*. *benthamiana* leaves infiltrated with pCamv35SGFP-annexin showed expected size of annexin-GFP fusion, which is larger than GFP (Fig 3B). This indicates that annexin-GFP fusion is intact fused.

### BSMV-HIGS of *Ha-annexin* causes impaired nematode parasitism

**Fig 4:**
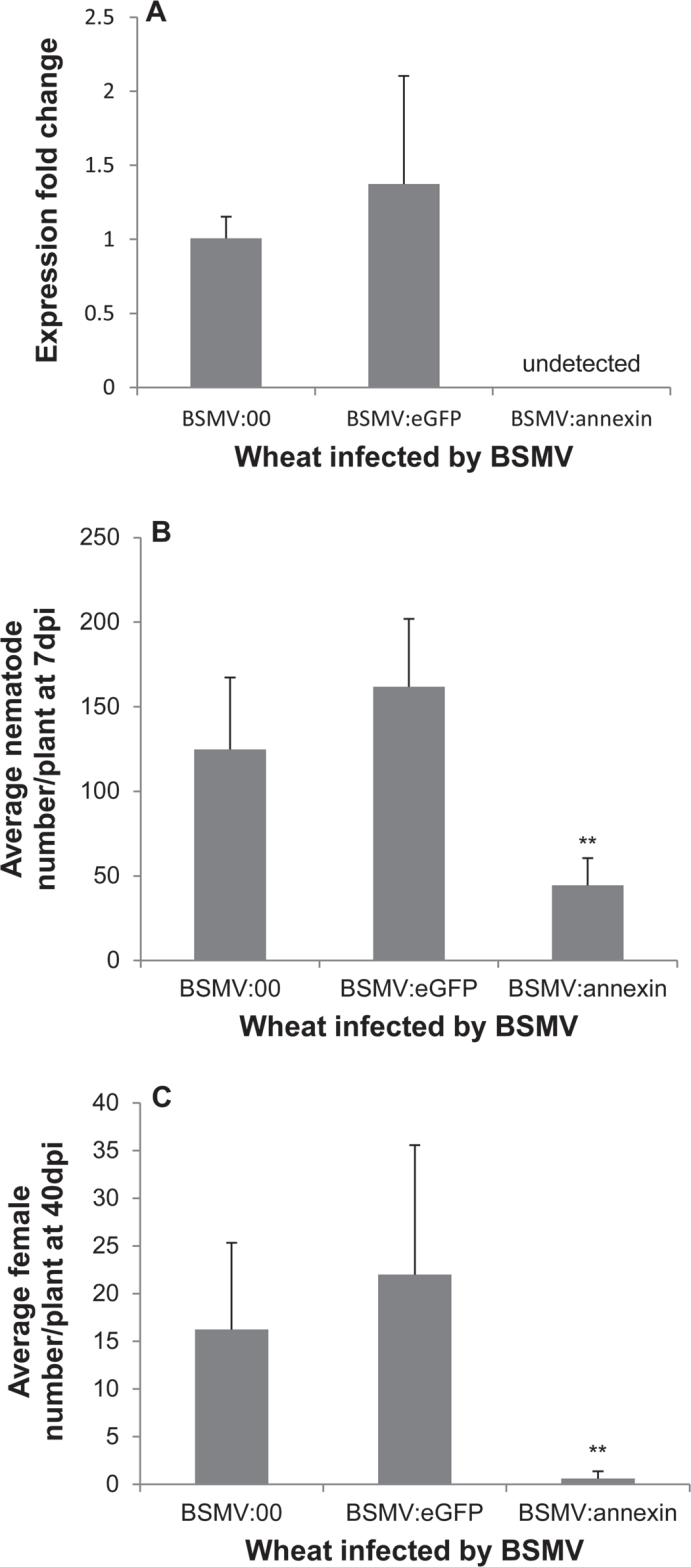
Effect of BSMV-HIGS of *Ha-annexin* on infection of wheat roots by *H*. *avenae*. (A) At 7 dpi, the expression of *Ha-annexin* in nematodes recovered from wheat inoculated by BSMV:*annexin* was not detected by qPCR with BSMV:00 and BSMV:*eGFP* as positive controls. Nematode infection of wheat inoculated by BSMV:*annexin* compared to the blank negative control BSMV:00 and the negative control BSMV:*eGFP* showed a highly significant reduction in the number of juveniles/plant at 7 dpi (B) and females/plant at 40 dpi (C) by Duncan test (*P*<0.01). Each column represents the mean with standard deviation.

In recent years, BSMV has become a popular vector for virus-induced gene silencing (VIGS) in wheat, and a BSMV-HIGS system has emerged [40]. We used this system to conduct a loss-of-function study of *Ha-annexin* in wheat, and two repetitive experiments yielded consistent results. This consistency demonstrated that the system worked well in silencing the expression of *Ha-annexin* (Fig 4A). The expression of *Ha-annexin* in nematodes recovered from wheat inoculated by BSMV:*annexin* was not detected, showing a significant reduction compared to that from BSMV:00 and BSMV:*eGFP* controls by qPCR (*P*<0.01) (Fig 4A). Additionally, due to the reduction in *Ha-annexin* expression, nematode infections of wheat inoculated by BSMV:*annexin* compared to the blank negative control BSMV:00 and the negative control BSMV:*eGFP* showed a highly significant reduction in the number of juveniles/plant at 7 dpi by 73% (Fig 4B) and females/plant at 40 dpi by 97% (Fig 4C) (*P*<0.01). Off-target silencing effects on other genes from *H*. *avenae*, BSMV and wheat should not happen because of the specificity of the selected fragment. Furthermore the BSMV infection symptom and root phenotype weren’t affected by the annexin silencing when compared to the controls. These results indicate that *Ha-annexin* plays important roles during the parasitism process at least in early stage of *H*. *avenae*.

### Ha-ANNEXIN suppresses plant defense

**Fig 5:**
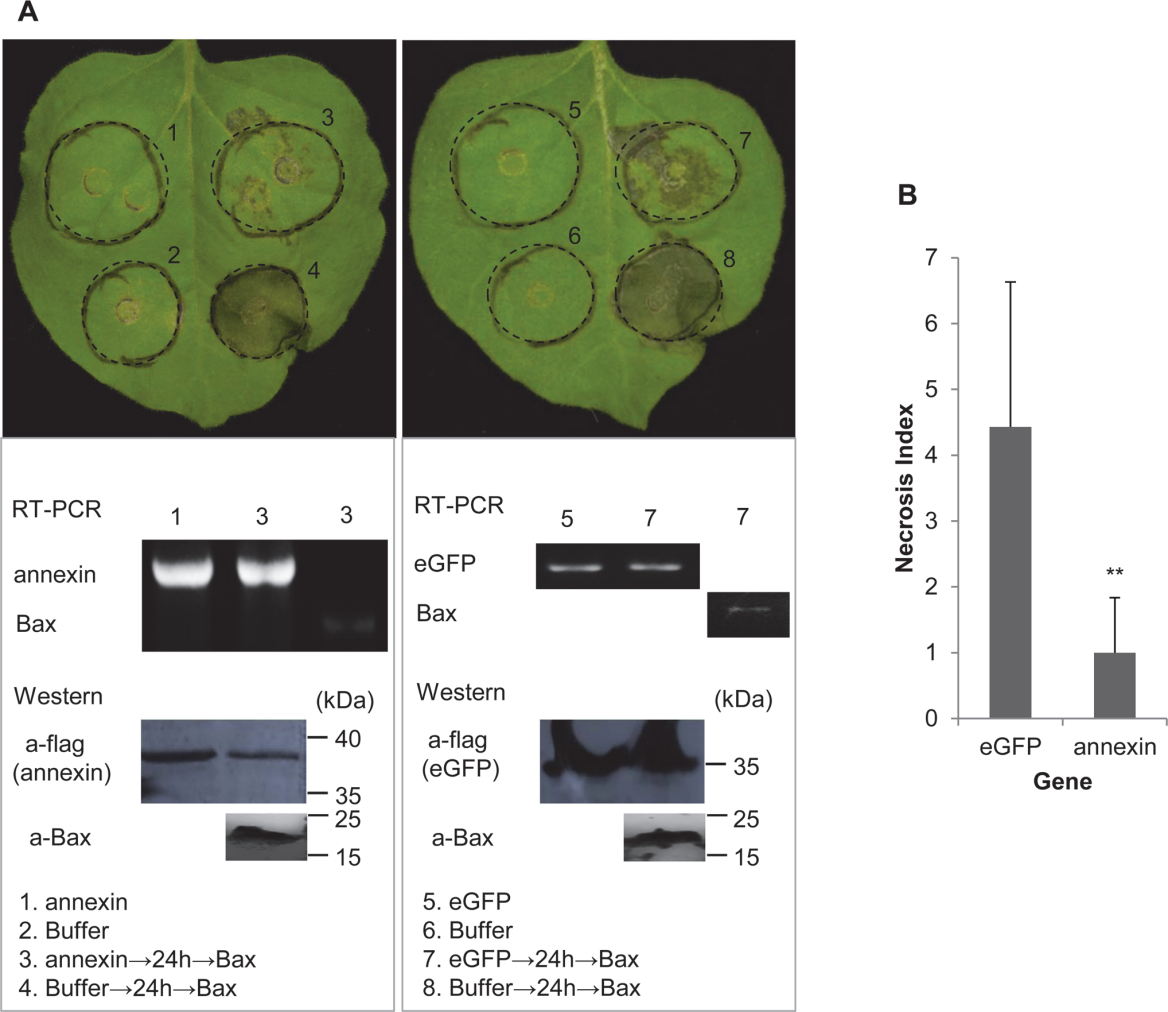
Suppression of BT-PCD by Ha-ANNEXIN. (A) Assay for the suppression of BAX-triggered cell death (BT-PCD) in *Nicotiana benthamiana* by Ha-ANNEXIN. *N*. *benthamiana* leaves were infiltrated with buffer or *Agrobacterium tumefaciens* cells containing a vector carrying the *Ha-annexin* gene or the negative control *eGFP* gene, either alone or followed 24 h later with *A*. *tumefaciens* cells carrying a mouse *Bax* gene. Photos of phenotypes of infiltrated leaves of *N*. *benthamiana* were taken approximately 7 days after the last infiltration. Results of the verification of gene expression of *Ha-annexin*, *eGFP* and *Bax* by RT-PCR or western blotting are shown below. (B) Necrosis Index of Ha-ANNEXIN and control eGFP followed by BAX. Each column represents the mean with standard deviation. The column with asterisks indicate a highly statistically significant reduction of the Necrosis Index of Ha-ANNEXIN compared with that of eGFP by t-test (*P*<0.01).

Two repetitive results of the BAX cell-death suppression assay in *N*. *benthamiana* were consistent; the infiltration spot of Ha-ANNEXIN followed by BAX was not as necrotic as that of eGFP followed by BAX (shown in representative photos, Fig 5A). RT-PCR and western blotting were conducted to verify the expression of *Ha-annexin*, *eGFP* and *Bax* from the transcriptional and translational levels, respectively. For a quantitative comparison, the degree of PCD (referred to as the “Necrosis Index”) of Ha-ANNEXIN and the eGFP control followed by BAX was scored and compared. As shown in Fig 5B, the Necrosis Index of Ha-ANNEXIN followed by BAX was low (0.9) compared with that of the negative eGFP control (4.1) (*P*<0.01). This finding suggested that Ha-ANNEXIN could suppress BT-PCD to some extent. Accordingly, ANNEXIN of *H*. *avenae* may play roles against plant defense.

**Fig 6:**
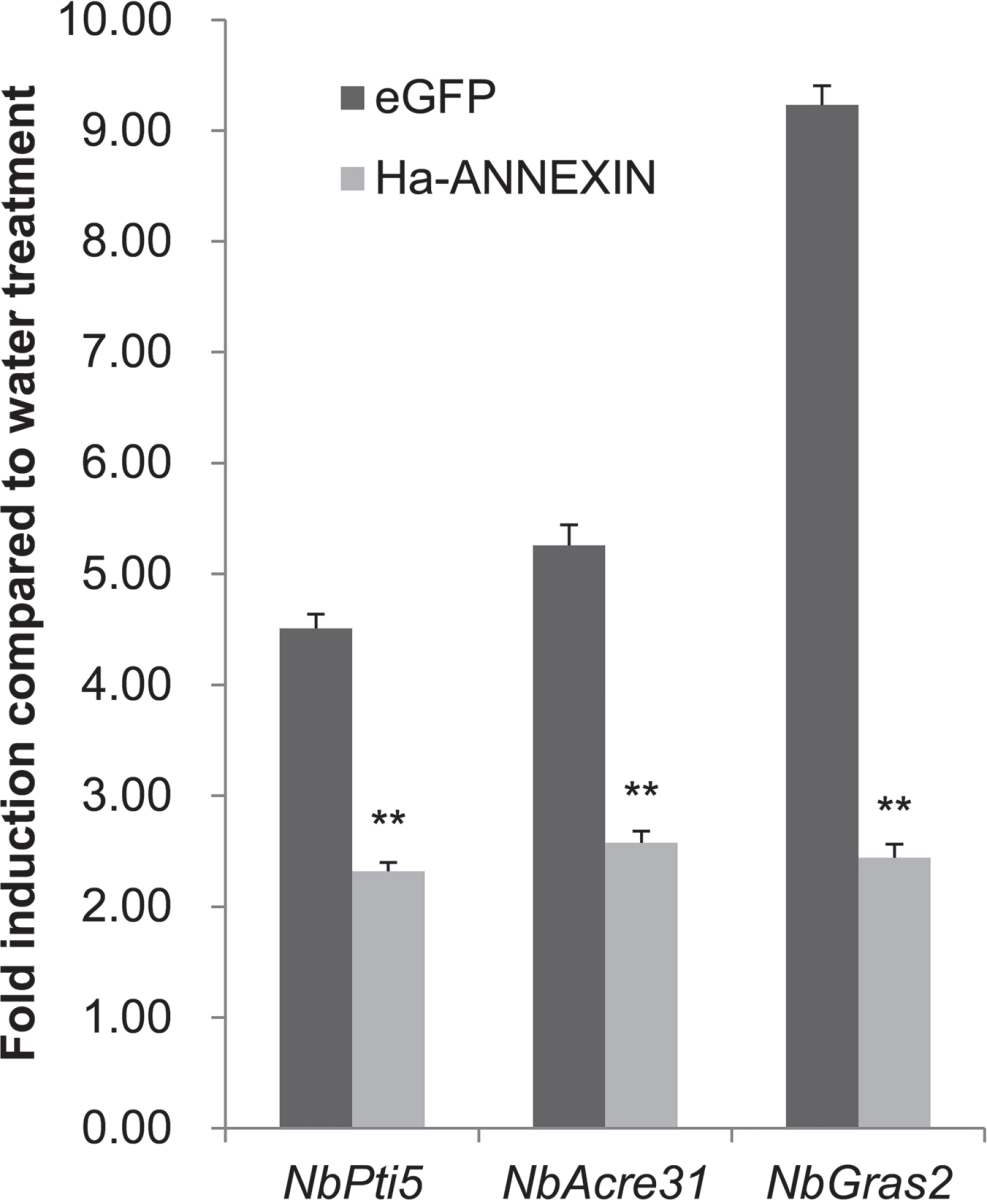
Ha-ANNEXIN suppresses flg22-triggered upregulation of PTI marker genes in *Nicotiana benthamiana*. The upregulation of three PTI marker genes—*NbPti5*, *NbAcre31* and *NbGras2*—after flg22 treatment in *N*. *benthamiana* leaf tissues expressing Ha-ANNEXIN or the negative control eGFP was compared using qPCR, respectively. Each column represents the mean with standard deviation. The column with asterisks indicate a highly statistically significant difference compared with the eGFP negative control by t-test (*P*<0.01).

To further determine whether ANNEXIN can suppress the PTI of plants, marker gene expression in *N*. *benthamiana* infiltrated with *A*. *tumefaciens* cells carrying *Ha-annexin* or control *eGFP* after flg22 treatment were compared. Two repetitive assays were conducted with consistent results and the results of one were shown in Fig 6. Three marker genes—*NbPti5*, *NbAcre31* and *NbGras2*—were induced by 1.3-, 1.6-, and 1.4-fold upregulation, respectively, in leaf tissues infiltrated by *A*. *tumefaciens* cells carrying *annexin*, in contrast to 3.5-, 4.3-, and 8.2-fold upregulation in *eGFP* control, respectively. An independent-samples t-test analysis showed that all three marker genes were significantly less upregulated in leaf tissues infiltrated by *A*. *tumefaciens* cells carrying *annexin* than in those with the *eGFP* control (*P*<0.01) (Fig 6). It means that Ha-ANNEXIN suppressed flg22-triggered PTI in *N*. *benthamiana*, *w*hich is one aspect of plant defense.

### Target point of Ha-ANNEXIN in PTI suppression

**Fig 7:**
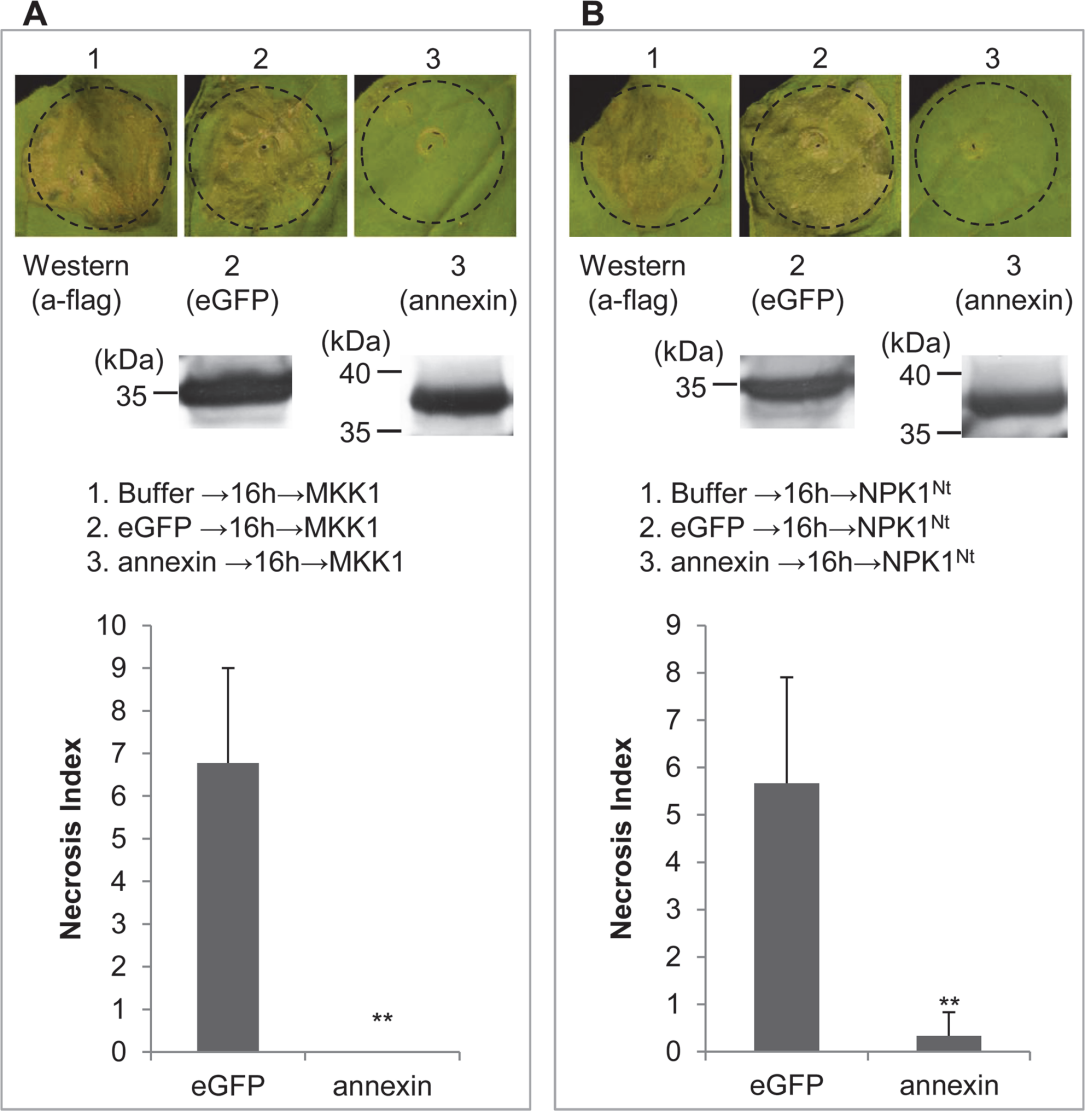
Ha-ANNEXIN can suppress MAPK-triggered cell death. Ha-ANNEXIN can suppress MKK1 (A)—or NPK1^Nt^ (B)-triggered cell death. Results of the verification of expression of *annexin and eGFP* by western blotting are shown below. The Necrosis Index of Ha-ANNEXIN and control eGFP followed by MKK1 (A) or NPK1^Nt^ (B) was scored. Each column represents the mean with standard deviation. The column with asterisks indicate a highly statistically significant reduction of the Necrosis Index of Ha-ANNEXIN compared with that of eGFP by t-test (*P*<0.01).

Plant MAPK cascades play pivotal roles in the PTI signaling pathway [23–27]. MAPK cascades consist of at least three protein kinases: a MAPK kinase kinase phosphorylates and activates a MAPK kinase, which subsequently activates a MAPK by phosphorylation [22]. To further study the mechanism of Ha-ANNEXIN in PTI suppression, we tested the suppression of PCD triggered by genes encoding MKK1 (a MAPK kinase) or the N terminus of NPK1 (residues 1 to 373; NPK1^Nt^) (a MAPK kinase kinase) when introduced by agroinfiltration into *N*. *benthamiana*. Two repetitive results of the cell-death suppression assay in *N*. *benthamiana* were consistent for both MKK1 and NPK1^Nt^. Western blotting was conducted to verify the protein expression of *Ha-annexin and eGFP* (Fig 7). As representative photos shown in Fig 7A, the infiltration spot of Ha-ANNEXIN followed by MKK1 was not as necrotic as that of eGFP followed by MKK1, which was the same as the assay for NPK1^Nt^ shown in representative photos in Fig 7B. Quantitatively, the Necrosis Index of Ha-ANNEXIN followed by MKK1 was low (0) compared with that of the negative eGFP control (6.8) (*P*<0.01) (Fig 7A), and the Necrosis Index of Ha-ANNEXIN followed by NPK1^Nt^ was also low (0.3) compared with that of the negative eGFP control (5.7) (*P*<0.01) (Fig 7B). The results revealed that Ha-ANNEXIN could suppress cell death triggered by the conditional expression of the MKK1 or NPK1^Nt^ in *N*. *benthamiana*, which means it is targeted at a point in the signaling pathway downstream of the two kinases MKK1 and NPK1.

## Discussion

In this study, we cloned an annexin-like gene from *H*. *avenae*. This gene encodes a protein with a significant similarity (up to 76%) to annexin 2, a secreted protein of *G*. *pallida* also without signal peptide [11]. There is a group of secretory proteins without signal peptides, which are exported by a non-classical secretion system independent of the classical ER-Golgi secretion pathway. There are increasing evidences about annexins without signal peptides that can be secreted. For example, human prostate gland selectively secretes high concentrations of annexin 1 through a highly selective mechanism that does not involve targeting to the endoplasmic reticulum by a hydrophobic signal sequence [41]. AtANN1 and AtANN2 from *Arabidopsis thaliana* have been predicted by SECRETOMEP software (http://www.cbs.dtu.dk/services/SecretomeP/) to be non-classical secreted proteins that can potentially become extracellular [42]. And they have been identified in the cell wall [43, 44] while ^15^N metabolically labelled AtANN1 has been found in washing fluid from leaves, suggesting an apoplastic location [45]. One annexin-like protein from plant pathogen *P*. *ramorum* [46] was identified as a cell-wall associated protein based on mass spectrometric sequence analysis of tryptic peptides obtained by proteolytic digestion of sodium dodecyl sulfate-treated mycelial cell walls. It’s an authentic secretory protein. Annexins in *C*. *elegans* and *G*. *pallida* are no exceptionally secreted, either. In *C*. *elegans*, annexins are expressed in a variety of tissues, including the gland cells of the terminal bulb of the esophagus, where they may have a role in exocytosis [47, 48]. gp-nex (annexin 2 from *G*. *pallida*) was isolated by screening a mixed stage *G*. *pallida* expression library using the monospecific polyclonal antibody IACR-PC320 which was raised using *G*. *rostochiensis* purified protein. The antibody is able to bind to excretory/secretory products from *G*. *pallida* second stage juveniles treated with the neurotransmitter 5 methoxy-N, N dimethyl tryptamine. It indicated that gp-nex is secreted out as one of the excretory/secretory products of *G*. *pallida*. Besides, it could be immunolocalised in the amphid, a nematode secretory organ [11]. *Ha-annexin* in our study is most related to gp-nex (annexin 2 from *G*. *pallida*) with 76% identities, which are higher than alignment with annexins of *C*. *elegans* (50% identities), *H*. *schachtii* (50% identities), and *H*. *glycines* (47% identities). So it’s reasonable for us to hypothesize that *Ha-annexin* has the secretory ability even without the classical secretion signal. And *in situ* hybridization of the gene showing the subventral glands localization (Fig 2B) further supported our hypothesis. Most of the effectors involved in parasitism are produced in the pharyngeal gland cells and are secreted into the host through the stylet [49]. qPCR analysis of *Ha-annexin* for the six developmental stages of *H*. *avenae* revealed that its expression was relatively higher in parJ2 (Fig 2C). So it’s most likely Ha-ANNEXIN mainly functions in the early parasitic stage. In addition, silencing of the gene *in vivo* using the BSMV-HIGS system caused significantly (*P*<0.01) impaired nematode infections at early parasitic stages (7 dpi) of wheat (Fig 4), which further confirmed that Ha-ANNEXIN played important roles during early parasitism.

Patel et al. reported that Hs4F01, an annexin-like effector from *H*. *schachtii*, interacted with an *Arabidopsis* oxidoreductase member of the 2OG-Fe(II) oxygenase family that was linked to host defense and stress response [13]. Recently, reports regarding nematode effectors suppressing plant defense are emerging [14–18]. We hypothesized that Ha-ANNEXIN functions in suppressing host defense. The ability to suppress BT-PCD in an agroinfiltration assay in *N*. *benthamiana* leaves has proven to be a valuable initial screening tool for pathogen effectors capable of suppressing defense-associated PCD [19–22]; therefore, we used this system. The assay of the transient expression of Ha-ANNEXIN in *N*. *benthamiana* suppressing BT-PCD confirmed our hypothesis. Furthermore, three marker genes of PTI in *N*. *benthamiana* were also suppressed by Ha-ANNEXIN. Our research gives direct verification that Ha-ANNEXIN can suppress plant defense and also confirms previous reports of Hs4F01 (an annexin from *H*. *schachtii*) linked to plant defense [13]. In addition, plant MAPK cascades play pivotal roles in the PTI signaling pathway by transducing signals from PRRs to downstream components [23–27]. We investigated if Ha-ANNEXIN involved in the MAPK signaling pathway. The results showed that Ha-ANNEXIN could suppress cell death triggered by the conditional expression of two kinases genes—*MKK1* and *NPK1*—in *N*. *benthamiana*, which encode a MAPK kinase and a MAPK kinase kinase that functions to transduce PAMP-triggered signals [22, 28–31], respectively. This finding suggested that *Ha-annexin* is targeted at a point downstream of the two kinases MKK1 and NPK1 of the MAPK pathway to suppress PTI. It is reported that plants often rely on elaborate signaling networks regulated by phytohormones to defend themselves from pathogen attack. And pathogens including plant-parasitic nematodes have adopted strategies to manipulate phytohormone-regulated plant defenses [50, 51]. While MAPK cascades play important roles in regulating plant defense hormone biosynthesis and signaling [23]. Previous report showed that Hs4F01 interacts with an oxidoreductase member of the 2OG-Fe (II) oxygenase family, which is linked to host defense and stress response and is involved in the biosynthesis and metabolism pathways of phytohormones [13, 52]. Therefore, further research should be conducted to investigate whether and how *Ha-annexin* regulates plant defense hormone biosynthesis and signaling downstream of MKK1 and NPK1 to modulate MAPK.

As direct genetics by the generation of mutant nematode lines for the knock-out of specific nematode genes is not feasible for plant parasitic nematodes at present, other strategies such as overexpressing effector proteins in the host plant and host-derived RNAi of specific effectors have been employed. However, genetic transformation of wheat is low efficacy and time consuming and *H*. *avenae* has a narrow host range, which limits the functional study of candidate effectors of the nematode. While VIGS, an alternate powerful tool to study the loss-of-function of target genes, lights the hope for us [40, 53, 54]. It is based on viruses activating the posttranscriptional gene-silencing defense response during the infection of plants [55]. In VIGS, a short sequence fragment of the gene of interest is inserted into a cloned virus plasmid, and the recombinant virus is then inoculated onto host plants. During multiplication and spread of the introduced virus, posttranscriptional gene silencing is triggered. The corresponding mRNAs of the targeted gene are selectively degraded to result in silencing of the gene [36, 40]. In recent years, BSMV, a Hordeivirus type member, has become a popular VIGS vector for the study of gene function in many monocots, including wheat [40]. Additionally, the BSMV-HIGS system has emerged for gene silencing in plant-associated organisms by the recombinant virus delivering double-stranded RNAs (dsRNAs) of targeted genes from host plants to pathogens. To our knowledge, only some researches on gene silencing of fungi are reported at present [40]. In one study [56], three predicted pathogenicity genes—a MAPK, a cyclophilin, and a calcineurin regulatory subunit of the wheat leaf rust fungus *Puccinia triticina*—were silenced by BSMV-HIGS, resulting in a disease-suppressed phenotype of *P*. *triticina*. The disease suppression indicated the likely involvement of these fungal genes in pathogenicity and demonstrated that BSMV-HIGS in plant-generated RNAi is an effective strategy for functional genomics in rust fungi. Our research here first introduces the system for function analysis of the nematode effector of *H*. *avenae*. The results of two repetitive experiments were successful and consistent. We demonstrated that the system worked well with a highly significant reduction in the expression level of *Ha-annexin* in nematodes compared to the controls (*P*<0.01) (Fig 4A). Consequently, nematode infections of wheat showed a highly significant reduction in the number of juveniles/plant at 7 dpi and females/plant at 40 dpi compared with the controls (*P*<0.01) (Fig 4B and 4C). These results demonstrated the pivotal roles *Ha-annexin* plays during the parasitism process at least early stage of *H*. *avenae*. Undoubtedly, BSMV-HIGS will be an important system for exploring the function of effectors of *H*. *avenae* in the future.

Furthermore, overexpressing effector proteins in the host plant is another important gain-of-function study strategy for nematode effectors. For example, *H*. *glycines HgSYV46* gene encodes a potentially secreted protein containing a CLE domain, and its overexpression in wild-type plants mimics expression of AtCVL3 (one CLE involved in maintaining the cell division/cell differentiation balance at the Arabidopsis apical meristem), arresting meristem development. This finding indicates that Hg-CLE might impact the cell division/differentiation of the syncytium initials, influencing feeding site development [57]. The BSMV-mediated overexpression of small heterologous proteins (BSMV-VOX), including pathogen effector proteins from plant-associated organisms in plants, has emerged [40]. For example, ToxA effector protein from *Pyrenophora tritici-repentis* was expressed using BSMV-VOX and induced cell death in wheat cultivars that are either sensitive or insensitive to the external application of ToxA. This finding confirms that the internal expression of ToxA in wheat induces cell death, regardless of a cultivar’s sensitivity to externally delivered ToxA [58]. Though with significant size constraint of BSMV-VOX currently, next-generation derivatives of BSMV vectors will allow stable expression of larger proteins in plants for functional analyses in the near future [40], which will be helpful for the further functional study of effectors of *H*. *avenae*, including *Ha-annexin*.

## Supporting Information

S1 Table List of primers used in this study. (DOCX)
