# Supplementary material for: An ANNEXIN-Like Protein from the Cereal Cyst Nematode Heterodera avenae Suppresses Plant Defense
Source: PLoS One. 2015 Apr 7;10(4):e0122256. doi: 10.1371/journal.pone.0122256 (PMC4388550; doi:10.1371/journal.pone.0122256)
Supplement: S1 Table — (DOCX) [file pone.0122256.s001.docx]

**S1 Table. List of primers used in this study.**

| **Primer name** | **Primer sequence (5'→3')** |
| --- | --- |
| 29D09-F | TCTCCAYCWTCCCAATTGTGT |
| 29D09-R | ACATSGCTCGGACTCGGCAC |
| annexin-S1 | GGACGGTTATTTGGCGGTGGTGGAA |
| annexin-R | TCCACTTCGCCCTCTT |
| annexin-A1 | TCTCGGCGATCTGTCGGTTGCTATGC |
| annexin-A2 | GGAAATTGGCGCTCACTTTTGGGTCG |
| annexinQCF4 | AATGATGTCTAACGCAA |
| annexinQCR4 | GTCGCTCAGTTCCCT |
| anne-qRT-S | ATCTTGGTCACGCATAGCA |
| anne-qRT-A | CTCCACTTCGCCCTCTT |
| GAPDH-qS1 | AGCGGCACAGAACATCATCC |
| GAPDH-qAS1 | GGTCCTCCGTGTAGCCCAAA |
| ann-*Xba* I-S | CGC*TCTAGA*ATGATGTCTAACGCAACC^a^ |
| ann-*Xho* I-AS | TAA*CTCGAG*GTTCCCTTTGATCAGTGT^a^ |
| ann-if-pCam35SGFP-S1 | *CCGGGGATCCTCTAGA*ATGATGTCTAACGCAACC |
| ann-if-pCam35SGFP-AS1 | *TCACCATGGTGTCGAC*GTTCCCTTTGATCAGTGT |
| ann-if107f-S1 | *TAGTGGATCCCCCGGG*ATGATGTCTAACGCAACC^b^ |
| ann-if107f-AS1 | *TTCATCGGCGGTCGAC*TCAGTTCCCTTTGATCAGTGT^b^ |
| *Sma*I-GFP-ORF | *cccggg*ATGGTGAGCAAGGGCGAG^a^ |
| GFP-ORF-*Sal*I | *GTCGAC*TTACTTGTACAGCTCGTCCATGC^a^ |
| MKK1-if-p107HA-S1 | *CACCAGCTAGCATCGAT*ATGGCTTTAGTCCGAG^b^ |
| MKK1-if-p107HA-AS1 | *TATGGGTACGCGGCCGC*CTTCAAATTACTCAG^b^ |
| NPK1(Nt)-if-p107HA-S1 | *CACCAGCTAGCATCGAT*ATGCAGGATTTCATCG^b^ |
| NPK1(Nt)-if-p107HA-AS1 | *TATGGGTACGCGGCCGC*GGTCCTAACATCCATC^b^ |
| *Cla*I-Bax-ORF | *ATCGAT*ATGGACGGGTCCGGGGAG^a^ |
| Bax-ORF-*Sal*I | *GTCGAC*TCAGCCCATCTTCTTCCAGATGG^a^ |
| eGFP-F1 | GACGTAAACGGCCACAAGTT |
| eGFP-F2 | CTCCAGCAGGACCATGTGAT |
| NbPti5-F | CCTCCAAGTTTGAGCTCGGATAGT [38] |
| NbPti5-R | CCAAGAAATTCTCCATGCACTCTGTC [38] |
| NbAcre31-F | AATTCGGCCATCGTGATCTTGGTC [38] |
| NbAcre31-R | GAGAAACTGGGATTGCCTGAAGGA [38] |
| NbGras2-F | TACCTAGCACCAAGCAGATGCAGA [38] |
| NbGras2-R | TCATGAGGCGTTACTCGGAGCATT [38] |
| NbEF1α-F | AAGGTCCAGTATGCCTGGGTGCTTGAC [39] |
| NbEF1α-R | AAGAATTCACAGGGAC AGTTCCAATACCA [39] |
| annG3-LIC-pBS-S1 | *AAGGAAGTTTAA*CAAAGAGGGCGAAGTGG^c^ |
| annG3-LIC-pBS-AS1 | *AACCACCACCACCGT*CTTCTCCAGCGGAGTGTT^c^ |
| eGFPG1-LIC-pBS-S1 | *AAGGAAGTTTAA*ACCCTCGTGACCACCCTGAC^c^ |
| eGFPG1-LIC-pBS-AS1 | *AACCACCACCACCGT*GTTCACCTTGATGCCGTTCT^c^ |

^a^Italicized letters are restriction sites.

^b^Italicized letters are sequences from the vector of pGR107 with flag-tag or HA-tag for cloning by In-Fusion Cloning technology.

^c^Italicized letters are adapter sequences for constructing targeted fragments into the pCaBS-γ vector using the LIC strategy [36].
